# Early Microglial Activation Following Closed-Head Concussive Injury Is Dominated by Pro-Inflammatory M-1 Type

**DOI:** 10.3389/fneur.2018.00964

**Published:** 2018-11-15

**Authors:** Sindhu K. Madathil, Bernard S. Wilfred, Sarah E. Urankar, Weihong Yang, Lai Yee Leung, Janice S. Gilsdorf, Deborah A. Shear

**Affiliations:** ^1^Brain Trauma Neuroprotection and Neurorestoration Branch, Center for Military Psychiatry and Neuroscience, Walter Reed Army Institute of Research, Silver Spring, MD, United States; ^2^Department of Surgery, Uniformed Services University of the Health Sciences, Bethesda, MD, United States

**Keywords:** microglia, inflammation, polarization, concussion, traumatic brain injury

## Abstract

Microglial activation is a pathological hallmark of traumatic brain injury (TBI). Following brain injury, activated microglia/macrophages adopt different phenotypes, generally categorized as M-1, or classically activated, and M-2, or alternatively activated. While the M-1, or pro-inflammatory phenotype is detrimental to recovery, M-2, or the anti-inflammatory phenotype, aids in brain repair. Recent findings also suggest the existence of mixed phenotype following brain injury, where activated microglia simultaneously express both M-1 and M-2 markers. The present study sought to determine microglial activation states at early time points (6–72 h) following single or repeated concussive injury in rats. Closed-head concussive injury was modeled in rats using projectile concussive impact injury, with either single or repeated impacts (4 impacts, 1 h apart). Brain samples were examined using immunohistochemical staining, inflammatory gene profiling and real-time polymerase chain reaction analyses to detect concussive injury induced changes in microglial activation and phenotype in cortex and hippocampal regions. Our findings demonstrate robust microglial activation following concussive brain injury. Moreover, we show that multiple concussions induced a unique rod-shaped microglial morphology that was also observed in other diffuse brain injury models. Histological studies revealed a predominance of MHC-II positive M-1 phenotype in the post-concussive microglial milieu following multiple impacts. Although there was simultaneous expression of M-1 and M-2 markers, gene expression results indicate a clear dominance in M-1 pro-inflammatory markers following both single and repeated concussions. While the increase in M-1 markers quickly resolved after a single concussion, they persisted following repeated concussions, indicating a pro-inflammatory environment induced by multiple concussions that may delay recovery and contribute to long-lasting consequences of concussion.

## Introduction

The prevalence of closed-head concussive injury is high in both military and civilian population. In fact, closed-head mild traumatic brain injury (mTBI) is the most common form, comprising 70–90% of all TBI incidence (1–3). While patients typically recover quickly from a single concussive injury, cumulative effect from multiple concussions can cause enduring damage that may evolve into neurodegenerative diseases (4, 5). Notably, athletes with a history of repeated concussions show late-life memory problems, psychiatric illness and increased risk of progressive neurodegenerative diseases such as Alzheimer's disease, and chronic traumatic encephalopathy (CTE) (4–7). Although, the association between repetitive TBI and the increased risk for CTE is well-recognized, the pathological mechanisms leading to neurodegeneration are poorly understood. Repetitive TBI in athletes is associated with chronic activation of microglia and appears to mediate pTau pathology and dementia in CTE (8). We have developed a rat model for closed-head mTBI called projectile concussive impact (PCI) injury that mimics several aspects of human concussive injury (9–11). Using this injury model, we have demonstrated acute increases in inflammatory cytokines, persistent gliosis, chronic functional neurological impairments, and white matter thinning following single or repetitive hits (9).

Neuroinflammation is considered as an important pathological mechanism leading to brain damage. Following physical trauma, inflammatory responses occur. These responses, such as the activation of microglia and macrophages, as well as the local release of inflammatory cytokines, have both positive and negative effects on the brain (12–14). Beneficial effects of inflammatory responses include wound healing and repair, but if left uncontrolled, inflammation can lead to neuronal damage and impede recovery. Persistent neuroinflammation has been observed years after a single TBI (15). Additionally, a recent positron emission tomography (PET)-study using translocator protein 18 kDa (TSPO), a marker of activated glial cell response, reported higher glial reactivity in retired football players, suggesting ongoing neuroinflammation that may contribute to later onset of neuropsychiatric problems (16). Microglia is rich in damage-associated molecular patterns (DAMPs) sensors and therefore responds rapidly to injury after detecting DAMPs (17). Once activated, microglia release cytokines, and chemokines that attract peripheral immune cells to infiltrate the brain parenchyma (18). Additionally, microglia responds to the inflammatory environment by changing their morphology and by assuming specific activation phenotypes (17). Similar to peripheral macrophages, microglia modify their activation state depending on stimuli. Two unique microglial polarization states have been discovered, the M-1 type (classical phenotype) and the M-2 type (alternative phenotype) (19). While M-1 are pro-inflammatory in nature and detrimental to recovery, M-2 acts as anti-inflammatory and supports tissue repair (19). M-1 microglia secretes high levels of IFN-γ, TNF-α, IL-1β, chemokines, and reactive oxygen species (20). The M-2 phenotype secretes neurotrophic factors and anti-inflammatory cytokines (IL-6, IL-10) and is sub-divided into M2a, M2b, and M2c depending on their specific phenotype markers, and are induced by different conditions or triggering factors (21). However, recent research findings question the microglial polarization concept. Histological and single-cell RNA-sequencing experiments have shown that microglia co-expressed the markers for both “polarized” states following TBI (22, 23).

Microglial activation, acute increase in cytokine production, and white matter abnormalities are the major pathological features found across various animal models of concussive injury (9, 24–27). Although inflammatory responses such as glial reactivity and increased cytokine production are studied following concussive injury, the role of different microglial activation states in the development of mTBI pathology and their association with injury severity are largely unknown. Here we examined whether single or repeated concussions can alter M-1 and M-2 activation states in different brain regions. Furthermore, the present study characterized acute changes in microglial morphology following concussion(s) using the PCI model. We used gene expression profiling in both the cortex and hippocampus to understand M-1 and M-2 marker expression following either single or repetitive injury. Immunohistochemical staining and quantification were used to determine the location and ratio of M-1 to M-2 microglia. We found that both single and repetitive concussions induced microglial activation and M-1 phenotype dominated over M-2 state. While a single concussion induced M-1 marker expression resolved quickly, multiple hits prolonged the pro-inflammatory cytokine gene expression. In addition, we observed unique morphological changes in cortical microglia following repeat hits. Overall, our findings support the hypothesis that concussive injury altered expression of M-1 and M-2 markers and provide a novel target for early therapeutic interventions.

## Materials and methods

### Animals

Male Sprague–Dawley rats (300−330 g, 6–8 weeks, Charles River Labs, Raleigh, VA) were used in all experiments. During the 1 week quarantine period, animals were housed in pairs and then housed individually. Animals were housed in a normal 12 h light/dark cycle with access to food and water *ad libitum*. The animal housing facility was accredited by the Association for Assessment and Accreditation of Laboratory Animal Care International. All animal procedures were approved by the Institutional Animal Care and Use Committee of Walter Reed Army Institute of Research (WRAIR). Animal research was conducted in compliance with the Animal Welfare Act and other federal statutes and regulations relating to animals and experiments involving animals, and adhered to the principles specified in the Guide for the Care and Use of Laboratory Animals, National Research Council (NRC) Publication, 2011 edition.

### Projectile concussive impact (PCI) model

**Figure 1:**
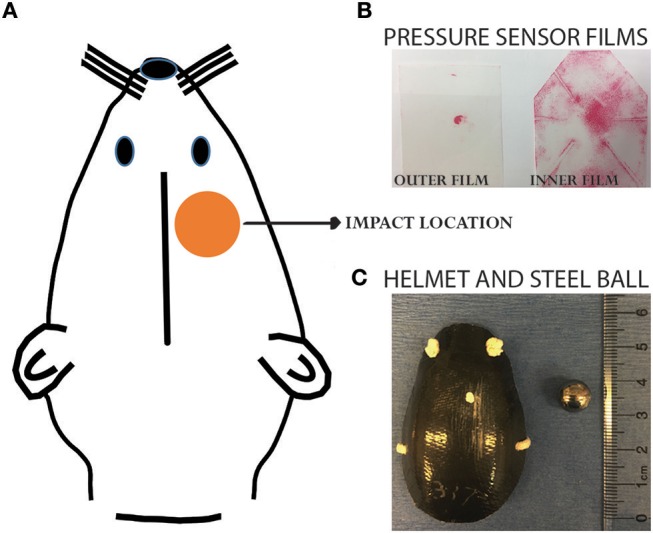
Projectile concussive injury model. **(A)** Illustration showing the impact location on a rat head. **(B)** Pressure sensor films following an impact. **(C)** Helmet and steel ball used for the impact. Please refer to our previous publications for more details of the PCI device (9, 11).

Animals were randomly assigned to four groups: single sham (SS), single concussion (SC), repeated sham (RS), and repeated concussion (RC). The SC group received one projectile impact, the RC group received 4 impacts spaced 1 h apart, and sham groups received the same procedures except projectile impact. Our previous studies have extensively characterized this closed-head concussive injury paradigm (9, 11). Briefly, rats were anesthetized with 4% isoflurane for 4 min in an induction chamber. A custom-designed carbon-fiber helmet (U.S Army Research Laboratory, Aberdeen Proving Ground, MD) was placed on the rat's head. Pressure sensor films (Fujifilm pre-scale pressure sensitive film) were adhered to the inner and outer surface of the helmet to record the distribution and magnitude of pressure from the impact (Figure 1). The rat was then placed on an elevated platform in a supine position with its head positioned above an oval opening in the platform. The projectile was made of a stainless steel ball (3.5 g weight and 10.05 mm diameter) placed in a tightly fit silicone tube directly beneath the oval opening of platform at a distance of 5 cm. A computer-controlled program was used to trigger the pressurized, rapid-release of projectile aiming at the right, dorsal–frontal quadrant of the brain (Figure 1). Following the impact, the helmet and sensor films were removed and the animal was returned to its home cage. Animals were euthanized at 6h and 72h after the final impact.

### Immunohistochemistry and cell counts

For immunohistochemistry processing, animals (*n* = 5–6 per time point/condition) were deeply anesthetized with intramuscular injection of ketamine/xylazine mixture (70 and 6 mg/kg, respectively,) and transcardialy perfused with saline followed by 4% paraformaldehyde (FD Neurotechnologies, Columbia MD). Whole brains were collected, post-fixed in 4% paraformaledyde and cryoprotected using 30% sucrose solution. Cryoprotected brains were shipped to FD Neurotechnologies for further processing and staining. Coronal free-floating sections (40 μm) were processed for immunohistochemical staining using specific antibodies for Iba-1 (1:1,000, Rb polyclonal, Wako Chemicals, Richmond, VA), CD163 (1:100, Ms monoclonal, Hycult Biotech Plymouth Meeting, PA) and MHC-II (1:100, Ms monoclonal, Abcam, Cambridge, MA). Secondary antibodies were conjugated with either Alexa-488 or Alexa-594. Images were captured using a BX51 microscope equipped with multichannel filters (Olympus, Waltham, MA). Co-localized cells (Iba-1/CD163 and Iba-1/MHC-II) from 6 sections/brain (400 μm apart) were counted from the cortex directly at 40X magnification using FITC/TRITC double filter.

### Inflammatory gene array and single tube real-time PCR (qRT-PCR)

For measuring inflammatory gene expression, a separate cohort of animals (*n* = 10 per group) which received sham procedure, single concussion or repeated concussions were used. At 6 h and 72 h post-injury, animals were deeply anesthetized with intramuscular injection of ketamine/xylazine mixture (70 and 6 mg/kg, respectively) and euthanized using a guillotine. Ipsilateral cortex and hippocampus were quickly dissected out and snap frozen using liquid nitrogen. Total RNA was isolated from snap-frozen ipsilateral cortex and hippocampal tissue using the mirVana total RNA isolation kit (Ambion, AM1560) in accordance to the manufacturer's instructions. RNA concentration and quality were determined using NanoDrop Lite (Thermo Scientific, Pittsburg, PA). Extracted RNA was immediately used to synthesize cDNA. One microgram (1 μg) RNA was reverse transcribed using TaqMan Reverse Transcription reagents (Applied Biosystems, Carlsbad, CA). For custom designed TaqMan inflammatory profiling arrays, equal volumes of cDNA from each sample were pooled and run on a single 96 well PCR-array plate per cohort (e.g., SC 6 h, RC 6 h). M-1 and M-2 specific genes included in the profiling arrays were chosen based on literature evidence (22, 28–30). Selected genes from the profiling arrays were later validated using individual sample qRT-PCR. Amplifications of these gene transcripts were carried out in triplicate using TaqMan universal PCR Master Mix on an ABI Step One Plus real time PCR machine (Applied Biosystems). Gene expression was normalized to the endogenous control, succinate dehydrogenase complex flavoprotein subunit A (Sdha) and the fold change per condition was calculated using the 2^−ΔΔ*Ct*^ (Ct is the threshold cycle) method, compared to the sham group.

### Data analysis

Target genes of interest were determined by examining PCR-array. Genes were classified as either upregulated or downregulated when the change in expression relative to sham was greater or equal to +1.5 fold or less than or equal to −1 fold, respectively. To generate heat maps, changes in individual gene expression levels were expressed as fold change over respective sham at corresponding time points and injury condition. qRT-PCR data and immunostained cell counts were analyzed using one-way ANOVA followed by Tukey's multiple comparisons test using GraphPad Prism version 7 software (La Jolla, CA). All data are presented as mean + SEM and for all comparisons a *p*-value <0.05 was considered significant.

## Results

### Concussive injury altered microglial morphology and Iba-1 gene expression

**Figure 2:**
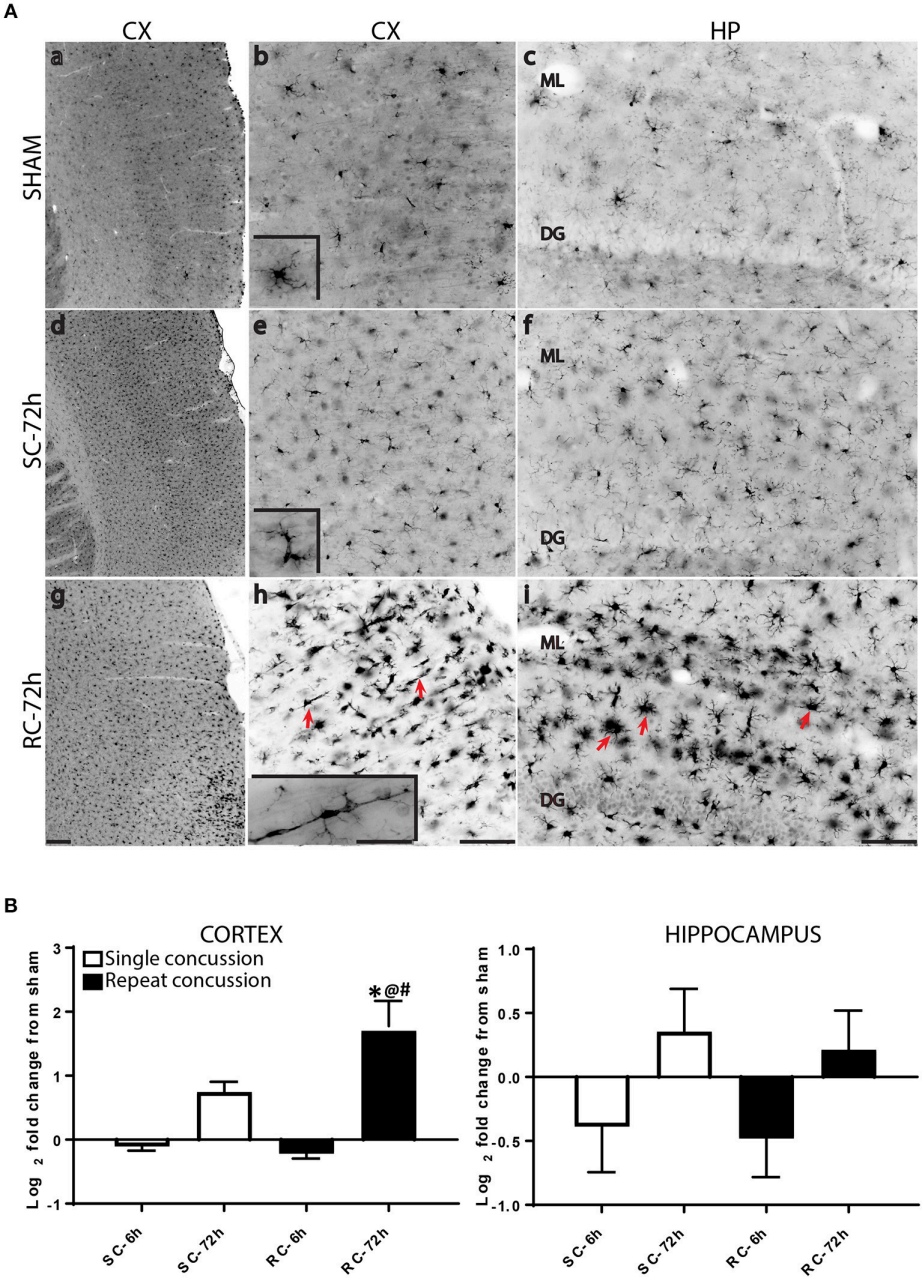
Changes in microglial morphology and Iba-1 expression following concussive injury. **(A)** Iba-1 immunohistochemical staining in the ipsilateral cortex and hippocampus at 72 h following either single or repeated concussion. Resting, ramified microglia was observed in the sham cortex and hippocampus (top panel, **a–c**). Following single hit, microglia showed activated morphology with retracted processes (middle panel, **d–f**). Repeated hits showed elongated microglia (**g,h**, arrows) in the cortex and bushy, hypertrophied microglia (**i**, arrows) in the hippocampus. **(B)** Quantification of Iba-1 gene expression in the cortex and hippocampus. One-way ANOVA with Tukey's *post-hoc* test. *p < 0.05 compared to single hit 6 h. ^@^*p* < 0.05 compared to repeated hit 6 h. ^#^*p* < 0.05 compared to Single hit 72 h. SC: single concussion, RC: repeated concussion, CX: cortex, HP: hippocampus, ML: molecular layer, DG: dentate gyrus. Scale bar in g (for left panel) = 200 μm, in h (middle panel) and I (left panel) = 100 μm and inset (for insets in middle panel) in h = 50 μm.

**Figure 3:**
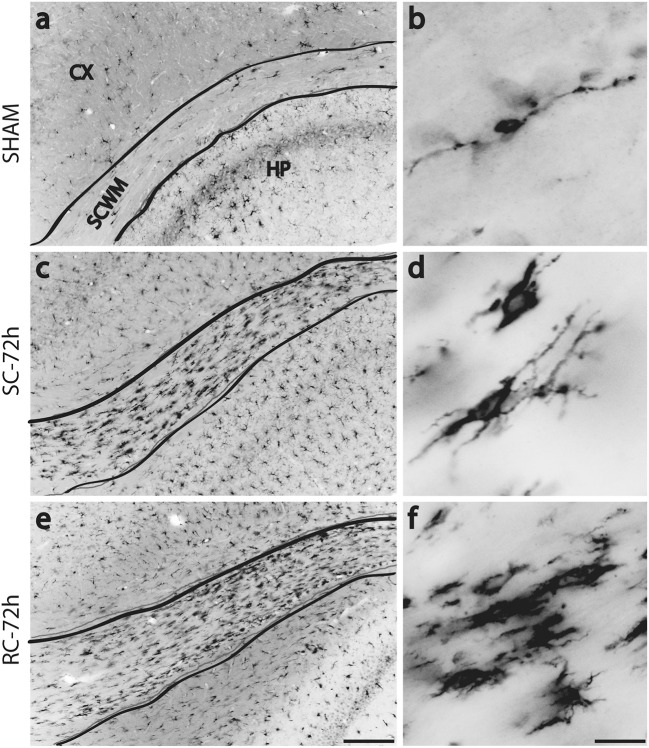
Changes in Iba-1 immunoreactivity in sub cortical white matter (SCWM) at 72 h post injury. Few scattered microglia with migrating morphology (long processes) were found in sham rat's SCWM **(a,b)**. Numerous, hypertrophied microglia were found in the SCWM following either single **(c,d)** or repeated concussions **(e,f)**. SC: single concussion, RC: repeated concussion, CX: cortex, HP: hippocampus, SCWM: subcortical white matter. Scale bar in e (for left panel) = 200 μm and in f (for right panel) = 50 μm.

While resting microglia has a ramified morphology, activation is characterized by a hypertrophied, bushy phenotype. We observed ramified microglia following sham injury (Figure 2A top panel) and activated phenotypes following either single (Figure 2A middle panel) or repeated concussion (Figure 2A, bottom panel). Although mild microglial reactivity was observed at 6 h post-injury, microglial morphological change was clearly noticeable at 72 h after repeated hits in entorhinal cortex, hippocampus and sub-cortical white matter (SCWM). While cortical microglia retracted processes following a single concussion (Figures 2A,d,e), multiple concussions induced both hypertrophied and elongated, radial microglia in the entorhinal cortex (Figures 2A,g,h). Some of these elongated radial microglia were seen coupled together to form train-like elongated structures (Figures 2A,h-inset). Hypertrophied bushy microglia were seen in both hippocampus (Figures 2A,i) and SCWM (Figure 3) following concussive injury.

We also quantified Iba-1 mRNA levels in both the cortex and hippocampus, using qRT-PCR. While cortical Iba-1 gene expression mirrored the histological observations, hippocampal Iba-1 gene expression showed a modest increase at 72 h following concussive injury (Figure 2B).

### Concussive injury altered inflammatory gene expression profile in the cortex and hippocampus

**Figure 4:**
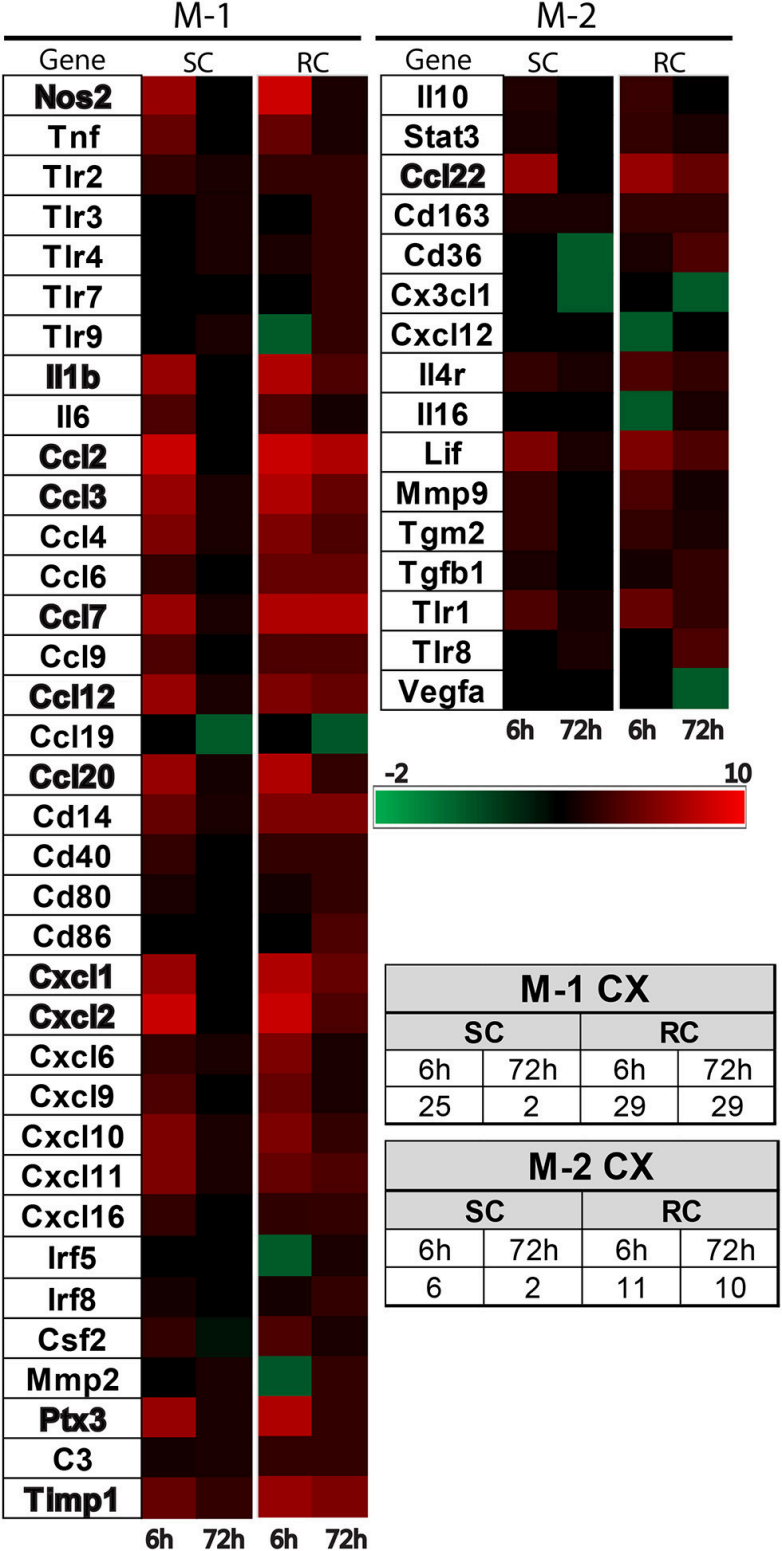
Heat map showing inflammatory gene profile of the ipsilateral cortex following concussive brain injury. Custom designed qRT-PCR mini-array identified genes that were upregulated (Red, ≥1.5 fold positive changes compared to sham group) or downregulated (Green, ≥1 fold negative change compared to sham group). Left panel shows altered M-1 markers and right panel shows altered M-2 markers. Names of the genes that were expressed more than 6 fold are identified in bold. The table insert shows the total number of genes that were changed under each condition. SC: single concussion, RC: repeated concussion, CX: cortex.

Inflammatory gene profiling was performed separately in cortex and hippocampus tissue using a custom designed PCR-array plate. Based on literature searches, we included 86 total M-1 and M-2 targets and 2 housekeeping genes (Table 1) in the PCR-array. Comparative analysis revealed 36 M-1 markers and 16 M-2 markers that were altered relative to their respective sham groups in the ipsilateral cortex (Figure 4). Single hit induced a brief upregulation of 25 M-1 related targets evident at 6 h post-injury, most of which returned to sham levels by 72 h post-injury, except for tissue inhibitor of metalloproteinases-1 (Timp1) (Figure 4). The only M-1 target that showed a downregulation at 72 h after a single hit was Chemokine (C-C motif) ligand 19 (Ccl19). Following repeated hits, 29 M-1 genes in the ipsilateral cortex were altered at 6 h post-injury. In contrast to the profile after a single hit, most of these genes remained altered at 72 h post-injury, including the downregulation of Cc119, indicating a persistent response following repeated injuries. Although fewer number of altered M-2 markers were detected compared to M-1 markers, M-2 targets in the cortex also exhibited a similar pattern with prolonged expression shifts following repeated hits than a single concussion (Figure 4). While several of M-1 markers showed expression levels higher than 6 fold, only Ccl22 among M-2 markers was upregulated 6 fold, indicating a clear dominance in M-1 marker upregulation following concussion (Figure 4).

**Table 1:** Gene table for inflammation profile array genes and their TaqMan IDs.

Rn00691090_m1	Rn01464736_g1	Rn00584362_m1	Rn00586403_m1	Rn01483988_g1	Rn01762214_m1	Rn01412404_m1	Rn00567818_m1	Rn00671924_m1	Rn00709368_m1	Rn01525859_g1
**Arg1**	**Ccl3**	**Cd40lg**	**Cxcl2**	**Il10**	**Irf8**	**Slamf6**	**Bmp2**	**Ccl4**	**Cd80**	**Tnf**
Rn00587615_m1	Rn00676341_m1	Rn00583505_m1	Rn01752376_m1	Rn00579590_m1	Rn00571654_m1	Rn00689963_g1	Rn00680715_m1	Rn00567011_m1	Rn01505881_m1	Rn00569995_m1
**Il13**	**Jak2**	**Stat1**	**Ccl1**	**Ccl5**	**Cd86**	**Il15**	**Stat3**	**Stat5a**	**Stat6**	**Ccl11**
Rn01467286_m1	Rn01471276_m1	Rn01464638_m1	Rn01536936_g1	Rn01439563_m1	Rn01400117_g1	Rn00580555_m1	Rn01536591_m1	Rn01481451_m1	Rn01403352_m1	Rn00572656_g1
**Ccl7**	**Ccl9**	**Ccl12**	**Ccl17**	**Ccl19**	**Ccl20**	**Ccl2**	**Ccl22**	**Ccl24**	**Ccl25**	**Cd14**
Rn00580728_m1	Rn01423584_g1	Rn00593186_m1	Rn00578225_m1	Rn01413889_g1	Rn00788261_g1	Rn00573260_m1	Rn01441840_m1	Rn01496393_m1	Rn00595504_m1	Rn00569848_m1
**Cd36**	**Cd40**	**Cx3cl1**	**Cxcl1**	**Cxcl10**	**Cxcl11**	**Cxcl12**	**Cxcl14**	**Cxcl16**	**Cxcl9**	**Tlr4**
Rn04224332_u1	Rn01640054_m1	Rn00678341_g1	Rn01441684_s1	Rn00484683_m1	Rn00569873_m1	Rn00594078_m1	Rn01477715_m1	Rn00580432_m1	Rn04244818_m1	Rn01759835_m1
**Tlr8**	**Tlr9**	**Dusp1**	**Fpr1**	**Gata3**	**Gata6**	**Ifng**	**Il16**	**Il1b**	**Il25**	**Il33**
Rn01410330_m1	Rn01435145_m1	Rn01500522_m1	Rn00573491_g1	Rn01408838_m1	Rn00821234_g1	Rn01538170_m1	Rn00579162_m1	Rn00561646_m1	Rn00440945_m1	Rn01769850_m1
**Il6**	**Irf4**	**Irf5**	**Lif**	**Marco**	**Mif**	**Mmp2**	**Mmp9**	**Nos2**	**Pparg**	**Ptx3**
Rn00571440_m1	Rn01430873_g1	Rn00441826_m1	Rn04181452_s1	Rn02133647_s1	Rn01488472_g1	Rn01507024_m1	Rn01471506_m1	Rn00573587_g1	Rn01767369_m1	Rn01456850_m1
**Tgm2**	**Timp1**	**Timp3**	**Tlr1**	**Tlr2**	**Tlr3**	**Il4r**	**Kdm6b**	**Cxcl6**	**Cd209a**	**Csf2**
Rn01511602_m1	Rn01490246_m1	Rn00566466_m1	Rn01414231_m1	Rn01456400_m1	Rn01492519_m1	Rn01771083_s1	Rn01456866_m1	Rn00572010_m1	Rn00590475_m1	Rn99999916_s1
**Vegfa**	**Chit1**	**C3**	**Cxcl3**	**Ccl6**	**Cd163**	**Tlr7**	**Il4**	**Tgfb1**	**Sdha**	**Gapdh**

*Bold items indicate gene name, followed by their respective ID*.

**Figure 5:**
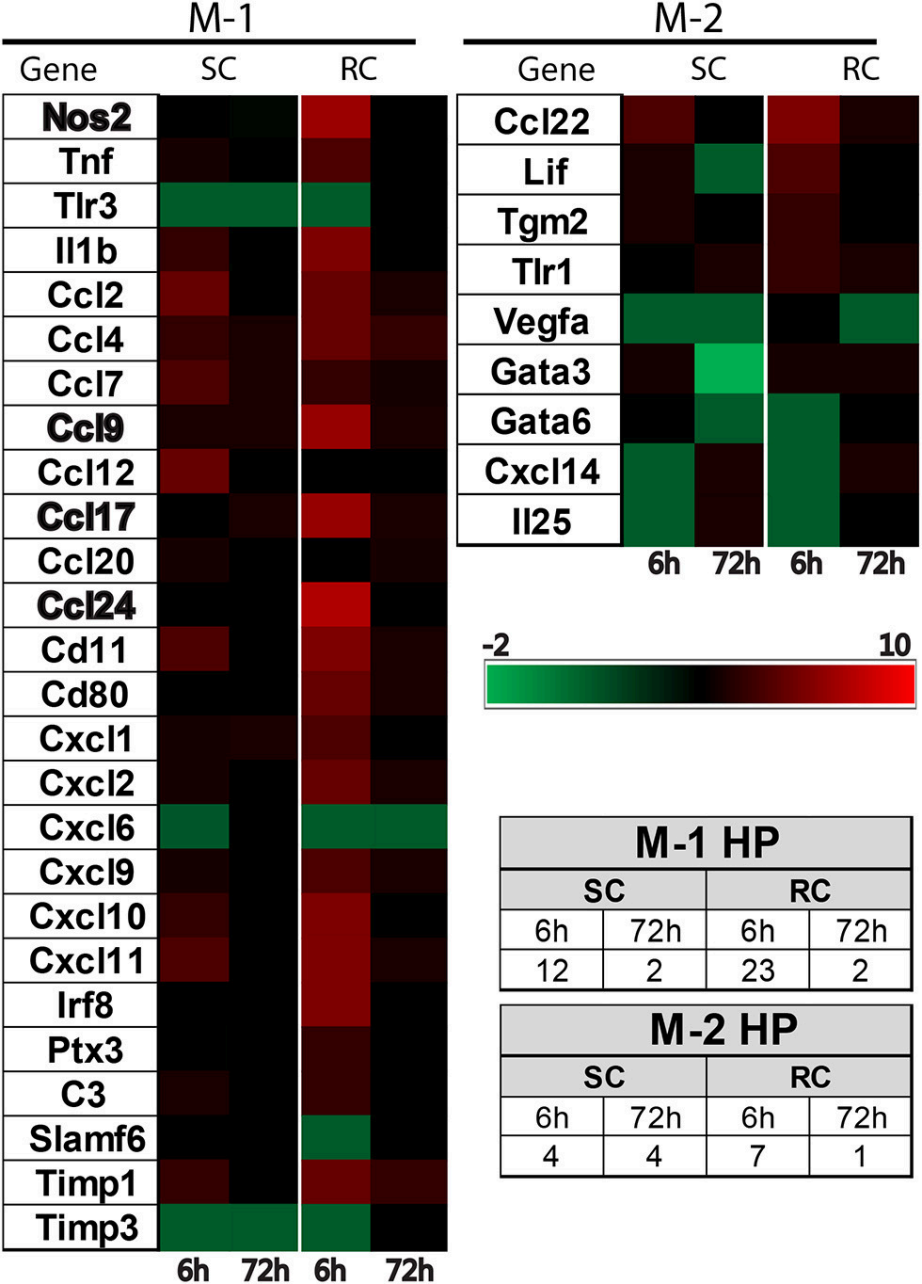
Heat map showing inflammatory gene profiling of the ipsilateral hippocampus following concussive brain injury. Custom designed qRT-PCR mini-array identified genes that are upregulated (Red, ≥ 1.5 fold positive change compared to sham group) or downregulated (Green, ≥ 1 fold negative change compared to sham group). Left panel heat map shows altered M-1 markers and right panel shows M-2 markers that were changed. Names of the genes expressed more than 6 fold are identified in bold. Table insert shows the total number of genes that are changed under each condition. SC: single concussion, RC: repeated concussion, HP: hippocampus.

Both single and repeated concussions also induced a transient change in M-1 marker expression in the hippocampus (Figure 5). Comparative analysis revealed that 25 M-1 markers and 9 M-2 markers were altered relative to sham expression levels in the ipsilateral hippocampus (Figure 5). Most of the hippocampal M-1 targets were upregulated except for downregulation of Tlr3, Timp3, and Cxcl6 after concussion. The expression patterns of several M-1 markers, including Nos2, Ccl2, and IL1b, were similar between cortex and hippocampus. The hippocampal M-2 markers were mostly downregulated (example: VEGF, Gata3, IL25) except for Ccl22, which was upregulated as observed in the cortex following concussion (Figure 5). Interestingly, the M-1 target, Timp1, is the only common gene that was upregulated in all four conditions (single and repeated concussions at 6 and 72 h) in both the cortex and hippocampus.

**Figure 6:**
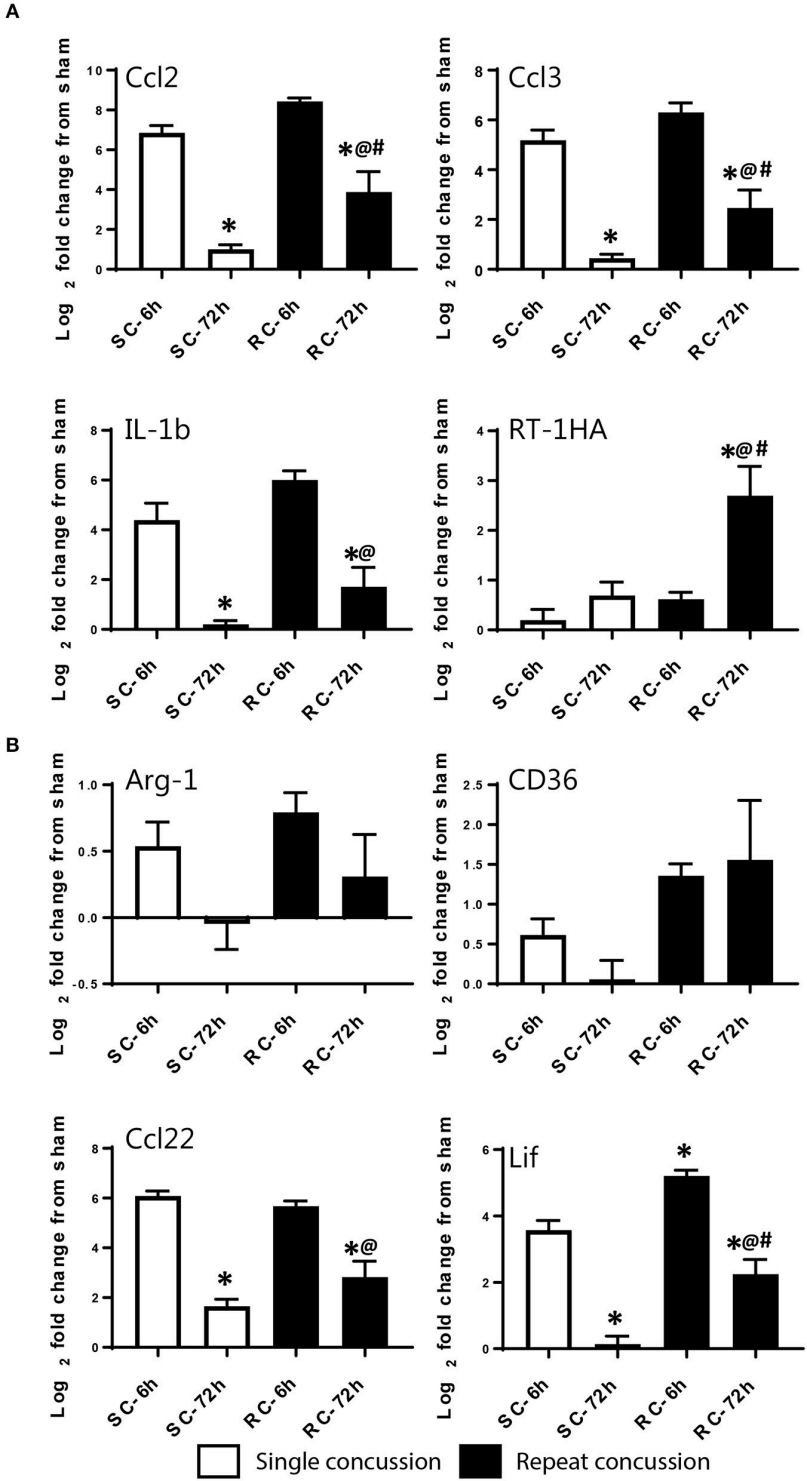
Single gene qRT-PCR of selected targets in the ipsilateral cortex. Selected gene targets including both **(A)** M-1 and **(B)** M-2 markers are run separately using individual samples (*n* = 10/condition). RT-1HA was included as this is part of MHC-II complex. One-way ANOVA with Tukey's *post-hoc* test. ^*^p<0.05 compared to single hit 6 h. ^@^*p* < 0.05 compared to repeated hit 6 h. ^#^*p* < 0.05 compared to Single hit 72 h. SC: single concussion, RC: repeated concussion, CX: cortex.

**Figure 7:**
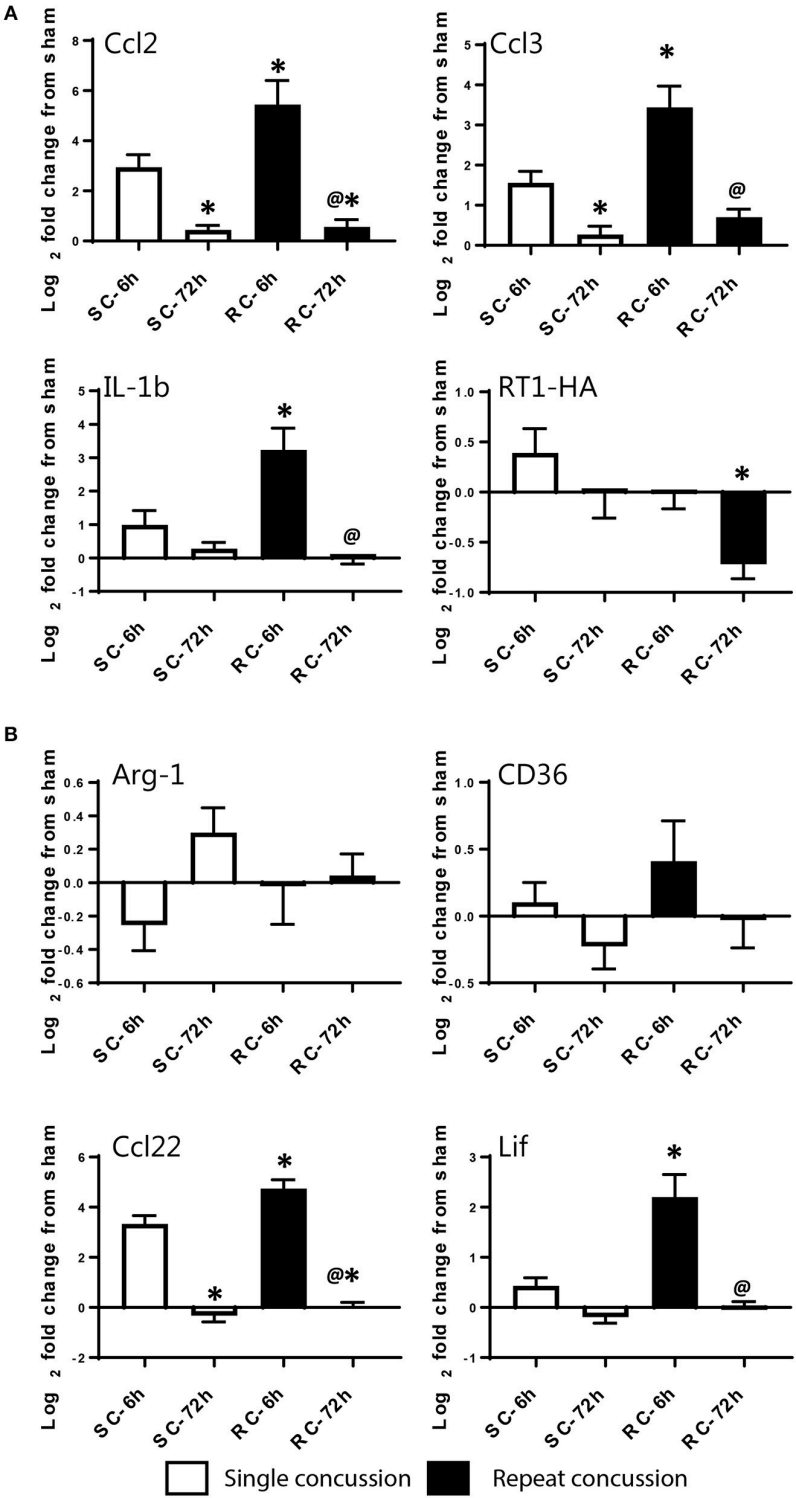
Single gene qRT-PCR of selected targets in the ipsilateral hippocampus. Selected gene targets including both **(A)** M-1 and **(B)** M-2 markers are run separately using individual samples (*n* = 10/condition). RT-1HA was included as this is part of MHC-II complex. One-way ANOVA with Tukey's *post-hoc* test. *p < 0.05 compared to single hit 6 h. ^@^*p* < 0.05 compared to repeated hit 6 h. SC: single concussion, RC: repeated concussion, HP: hippocampus.

We validated the expression levels of selected targets using individual qRT-PCR in both the cortex and hippocampus (Figures 6, 7). Target TaqMan array IDs are given in supplementary Table 1. We have also included RT-1HA, a component of MHC-II complex (Figures 6A, 7A) and Arg-1, a well-characterized M-2 marker previously shown altered expression following TBI (Figures 6B, 7B). Our individual RT-PCR runs echoed the inflammatory array pattern demonstrating a predominance for M-1 related gene upregulation following concussive injury.

### Pro-inflammatory M-1 type cells predominates microglial milieu following concussive injury

**Figure 8:**
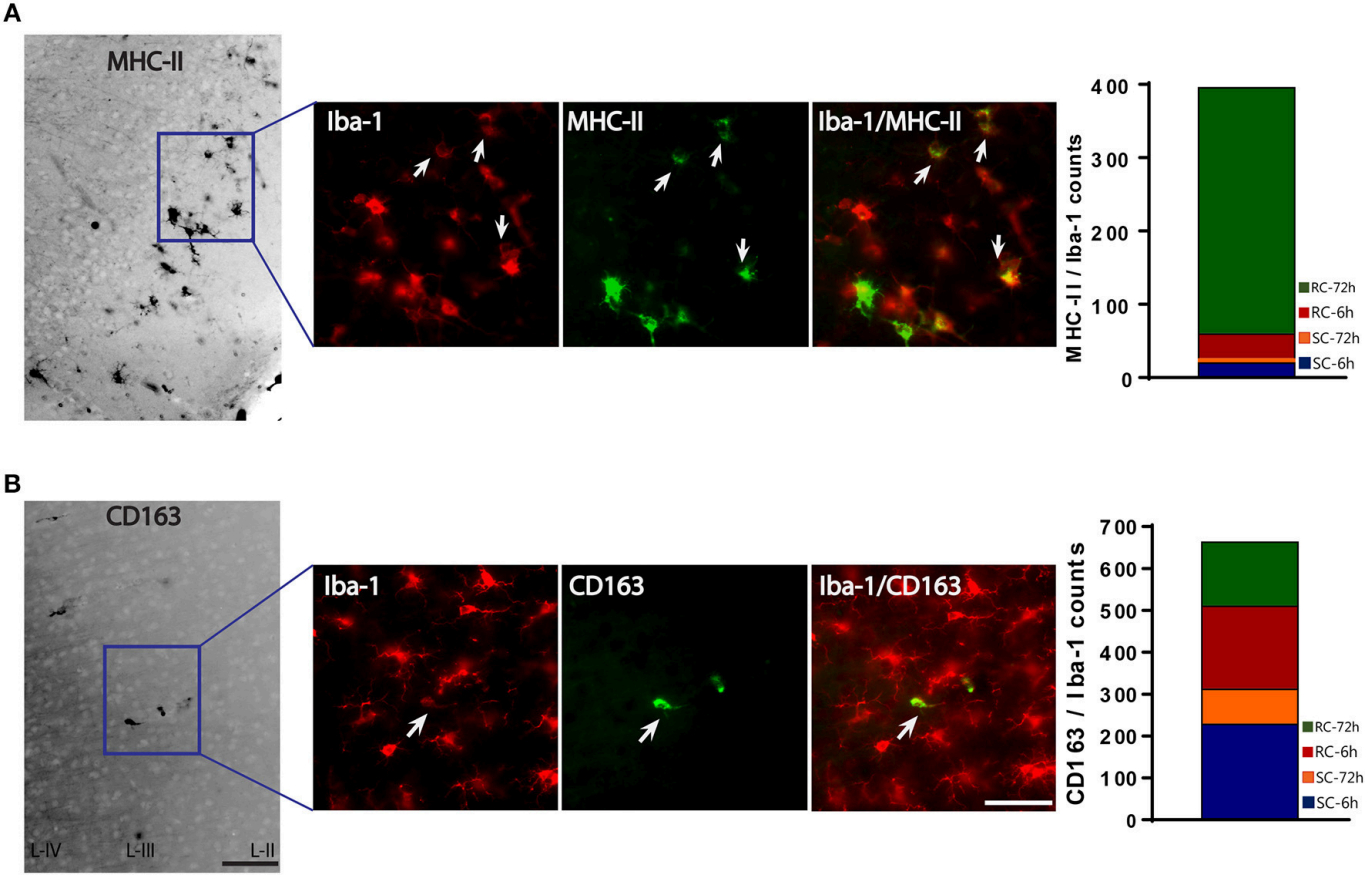
M-1 and M-2 marker expression by activated microglia in the ipsilateral cortex. **(A)** MHC-II (M-1 marker) stained microglia co-labeled with Iba-1 were found mainly in the entorhinal cortex. Co-localized cells were counted from entire cortex. **(B)** CD163 (M-2 marker) stained microglia co-labeled with Iba-1. Co-localized cells were counted from entire cortex (bar graphs). Arrows indicate co-localized cells. Scale bar = 50 μm.

We used MHC-II/Iba-1 for M-1 and CD163/Iba-1 for M-2 microglial labeling. Although we did see gene expression changes following qRT-PCR in the hippocampus, only very few, scattered M-1 or M-2 positive cells were seen in the hippocampus following immunohistochemistry and therefore was not included in the counting. Co-labeled cells were counted directly from the ipsilateral cortex (6 sections/brain) using a double filter. While most of the MHC-II positive cells co-localized with Iba-1 microglia, there were cells that did not co-localize (Figure 8A). While few occasional MHC-II positive cells were observed following sham procedure or single concussion, repeated concussion induced robust expression of MHC-II (Figure 8A), mainly at 72 h post-injury. MHC-II positive glia (M-1) were mainly located in the entorhinal part of the cortex (Figure 8A). In contrast to MHC-II cells, CD163 positive M-2 cells were found throughout the cortex in both sham and injured rats. Although there were more CD163/Iba-1 positive cells (M-2) than MHC-II cells at 6 h post-injury, this anti-inflammatory M-2 expression appeared to be transient (Figure 8B).

**Figure 9:**
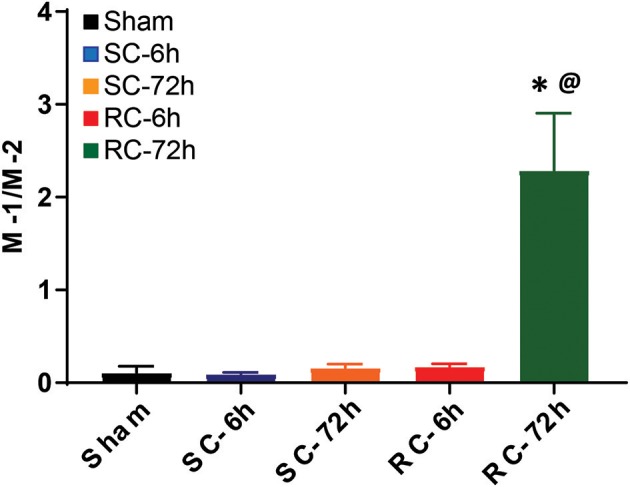
M-1 to M-2 microglial ratio in the ipsilateral cortex. M-1/M-2 ratio lower than 1 favored anti-inflammation, as observed in in sham group and in both SC and RC groups at 6 h. M-1/M-2 ratio >1 supported inflammatory environment, evident in RC group 72 h group. One-way ANOVA with Tukey's *post-hoc* test.*p < 0.05 compared to single hit 6 h. ^@^*p* < 0.05 compared to repeated hit. SC: single concussion, RC: repeated concussion.

The M-1/M-2 ratio can be used to evaluate pro- (above 1:1 ratio) or anti- (below 1:1 ratio) inflammatory environment.The M-1/M-2 ratio showed a significant increase at 72 h following multiple impacts (Figure 9) compared to sham and single concussion, indicating that a majority of the activated microglia following repeated impacts were pro-inflammatory. Low M-1/M-2 ratio indicating an anti-inflammatory environment was observed in sham rats, because of the presence of CD163 positive cells (Figure 9). Although single concussion and 6 h repeated concussion groups had low M-1/M-2 ratio, this was not significantly different from sham group (Figure 9).

## Discussion

The goal of the current study was to analyze early changes in microglial activation following single or repetitive closed-head concussive injury. Although the link between concussive injury and chronic neurodegeneration is well-established, early pathological changes following concussion is less explored. Increased glial reactivity (both microglia and astrocytes) was visualized by PET imaging in young, active NFL players indicating the onset of early neuroinflammation in the absence of gross morphological or functional changes (16). This highlights the importance of studying early changes following concussion, which may help to design therapeutics that could be administered before the onset of neurodegenerative pathology. We have previously reported increased levels of inflammatory cytokines in the cerebro-spinal fluid (CSF) and serum following repeated concussive injuries in rats (9). Cytokine-induced neutrophil chemoattractant 1 (CINC-1), TIMP-1 and L-selectin were increased as early as 1 h following multiple concussions (9). These cytokine changes in biofluids may reflect acute brain inflammation, as well as cytokine synthesis and secretion from reactive microglia/macrophages. To better understand the microglial changes in concussed brain, we performed a detailed phenotypic analysis of M-1 or M-2 like marker expression at 6 and 72 h following single and repeated concussions by using custom designed qRT-PCR array. We observed an increase in the expression of several M-1 targets and a few M-2 markers following both single and repeated hit injuries. mRNA levels of several cytokines including Ccl2, Ccl3, IL-1b, Ccl22, and Lif were found to be upregulated. While the increase was transient following single hit, it appeared to continue for days following repeat hits. Further, we found unique morphological changes in the microglia following repeated injuries. Collectively, these data suggest early microglial changes including both morphological and phenotypic alterations following concussive brain injury.

Microglia respond to the injury milieu in part through their morphology. Brain injury alters the activation state of microglia that is clearly characterized by distinct morphological features (28). Although morphology does not reliably reflect the functions or RNA expression profile phenotypes, it indicates that the cell is responding to altered homeostasis and researchers consider morphological change as an indication of neuroinflammation. Compared to healthy ramified microglia, reactive microglia comes in various shapes including amoeboid, hypertrophied and bushy (31). In our study, microglial morphological changes were more prominent following multiple impacts. By 72 h, many hypertrophied, bushy microglia were found in the cortex, hippocampus, and SCWM. This was not surprising, as active hypertrophied microglia has been observed in both single and repeated closed head injury (CHI) (24, 32, 33). However, we did not observe amoeboid microglia as opposed to focal TBI where amoeboid glial cells were found throughout the contused tissue (34). Besides the classical activation morphology, we also observed radial, train-like microglial formations in the entorhinal cortex following repeated hits. This distinct, rod shaped microglial morphology was previously reported following fluid percussion injury (FPI) in rats (35, 36). The exact function of these train-like glial formations are not yet known. In our histological studies, they did not co-localize with either M-1 marker MHC-II or M-2 marker CD163. However, this does not necessarily mean that they are not M-1 or M-2. It is possible that they may express other M-1 or M-2 targets. It appears that this interesting microglial shape is unique to diffuse type brain injury (35–37), suggesting that concussive injury produced by the PCI model shares pathological similarities with other diffused injury models.

Although shifts in microglial phenotypes are well-studied in moderate TBI using controlled cortical impact (CCI) or FPI models (22, 38–41), dynamics of microglial polarization is largely unknown following CHI. To our knowledge, only one study reported increased M-1 marker OX-6 or MHC-II staining in microglia like cells following closed head blast injury (42) that however did not examine multiple M-1 and M-2 markers to determine their polarization bias. In the present study, we designed a custom inflammation gene array plate which contained probes for known M-1 like and M-2 like markers (21, 22) in order to specifically profile inflammatory cytokines and chemokines in the brain following single and multiple concussive injuries. While several M-1 markers (Nos2, IL-1b, Ccl2, Ccl3, Ccl7, Ccl12, Ccl20, Cxcl1, Cxcl2, Ptx3, Timp1) showed significant upregulation, only a few M-2 markers (Ccl22, Lif) showed an increase, indicating the predominance of pro-inflammatory M-1 phenotype following concussion. These findings are akin to other TBI studies, where similar M-1 targets showed an increase following TBI (22, 40, 43). While focal TBI studies show changes in inflammation-related cytokine/chemokine expression lasting for weeks post TBI, in our model of single concussion, majority of the M-1 markers that upregulated at 6 h was resolved by 72 h. However, we observed persistent inflammatory response following multiple hits, indicating that the cumulative pathological response following repetitive injury took longer to resolve. Increased neuroinflammation as early as 24 h that lasted for weeks to years has been reported in preclinical studies of repetitive CHI (32, 44–46). Our previous study using PCI model also showed more robust pathology following multiple impacts. Compared to a single impact, acute increase in cytokine levels and GFAP expression were observed following repeated concussions (9–11). It is possible that this acute neuroinflammation may lead to long-lasting behavioral deficits. We have observed chronic functional impairment following both single and repeated impacts in the PCI model (9). Limiting acute neuroinflammatory responses may delay or halt chronic behavioral impairments that develop following concussion. Notably, acute administration of MW151, an inhibitor of brain proinflammatory cytokine upregulation have been shown to prevent chronic cognitive impairment following CHI in mice (37).

M-2 marker expression following concussion(s) in the PCI model differs from that of focal-TBI models. Our immunoprofiling did not show a change in M-2 marker Arginase-1 as it was found to be upregulated following focal TBI (22, 43, 47). In another study, Arginase positive M-2 microglia was detected following CCI injury and its number was increased by IL-2/anti-IL-2 complex treatment (26). It is possible that in our studies, PCI induced a more diffused pathology that is different from the focal-CCI injury model. Supporting this notion, Arginase-1 expression was not found to be altered after FPI in rats (35), a diffuse form of TBI. Arginase-1 is the arginine degrading enzyme that plays a role in anti-inflammation, cell survival and regeneration (48). Arginase-1 expression in microglia and macrophages are thought to drive them toward a M2 phenotype. However, flow cytometric analysis of isolated macrophage/microglia from injured brain demonstrated that 42% of Arginase positive cells co-expressed iNOS, a M-1 marker, indicating concurrent expression pattern of both M-1 and M-2 markers following TBI (40). Co-existence of M-1 and M-2 markers are reported in other TBI studies as well. Using a reporter mouse model for Arginase-1 expression, brain infiltrating macrophages following TBI identified as Arg1+ or Arg1–, revealed simultaneous gene expression of pro- and anti-inflammatory chemokines (49). Following severe CCI injury, a spectrum of macrophagic and microglial phenotypes were observed rather than showing a bias toward M-1 or M-2 (41). Mixed microglial phenotype with simultaneous expression of M-1 and M-2 markers were also reported following CCI in mice (22). In our concussion model, although we observed a mixture of M-1 and M-2 phenotypes, M-1 markers clearly dominated the acute injury environment. Both cortex and hippocampal regions showed similar M-1 response.

One of the highly expressed (>8) M-1 target was Nos2, the gene that codes for the enzyme inducible Nitric oxide synthase (iNOS) that is responsible for producing pathological form of nitric oxide (NO). Once activated, Nos2 can produce NO for hours to days contributing to oxidative damage to neighboring neurons (50). Acute increase in iNOS expression was observed in isolated microglia/macrophages following TBI (40) and inhibition of iNOS is shown to be neuroprotective following TBI (51). Another interesting neuroinflammatory marker upregulated across all conditions was TIMP-1. TIMP-1 is the inducible form of TIMPs that is known to be upregulated by inflammatory stimuli such as IL-1b and TNF-α (52, 53). Elevated TIMP-1 levels were observed in the CSF of TBI patients (54). Our previous study also demonstrated increased TIMP-1 levels in both serum and CSF following concussion (9). It is possible that the increase in TIMP-1 expression in the brain may have contributed to its elevation in biofluids. The elevated expression of M-1 markers such as Nos2 and TIMP-1 points to the presence of an early inflammatory environment following CHI. Interventions to curb M-1 phenotype expression may provide therapeutic benefits. Recent research on microglial phenotype emphasizes the therapeutic importance of shifting M-1 type to M-2 in reducing neuroinflammation. Treatments with Omega-3, HMGB-1 inhibitor glycyrrhizin, stem cell exosomes, atorvastatin and therapeutic hypothermia (55–58) were all shown to enhance M-2 polarization while reducing neuroinflammation following TBI.

We did not perform qRT-PCR studies using isolated microglia, and therefore, multiple cell types may have contributed to the cytokine/chemokine gene expression. To confirm the localization of M-1 and M-2 markers to microglia, we performed co-localization studies using microglial marker Iba-1. Quantification of MHC-II/Iba-1 (M-1 type) and CD163/Iba-1 (M-2 type) from cortical regions confirmed the RT-PCR results that showed a dominance in M-1 type following multiple hits at 72 h. However, at 6 h following single or repeated injuries, more CD163 cells were observed compared to MHC-II positive cells indicating more M-2 type cells than M-1 type at 6 h following concussion. Therefore, it appears that immediate post-injury environment is anti-inflammatory. However, the ratio of M-1/M-2 was not significantly different from sham following single concussion or 6 h repeated concussion. However, following focal-injury, transient upregulation of M-2 like phenotype later replaced by M-1 type was observed (40). At 6 h post-injury, the mRNA levels did not directly translate into the histological results. It is probable that the cell's defensive mechanisms to control neuroinflammation such as microRNAs that block inflammatory mRNAs are active immediately following a concussion. Interestingly, we previously observed increased mir-145 levels in circulation following concussive injury (9). In polarized microglia, mir-145 increase is strongly associated with M-2 phenotype (29), supporting our notion that microRNA-mediated regulatory mechanisms may be involved in favoring M-2 phenotype immediately after injury.

There are several limitations in the current study, particularly in examining microglial phenotypes following concussive injury. Although qPCR results show a clear dominance in pro-inflammatory markers, our study did not examine the source of these mRNAs. Repeated concussive injury is reported to have macrophage infiltration into the injured parenchyma (59) and it is likely that in our study macrophages contributed partly to the post-injury inflammation. Although we used Iba-1 immunohistochemistry to detect microglia, macrophages also express Iba-1 and may result in false positive staining. Recent research identified neurotoxic A1 astrocytes and inflammatory neurons that secrete cytokines and chemokines triggering secondary damage cascade (60, 61). To some extent they may be also responsible for the M-1/M-2 marker upregulation. Our study did not address the possibility of co-expression of M-1 and M-2 markers in the same microglia. On the other hand, we observed upregulation of both M-1 and M-2 markers indicating that inflammatory milieu following concussive injury was not exclusively M-1 or M-2 type microglia. Another limitation for the current study is the lack of functional correlates. Although the current study did not include any functional outcome metric, our previous study has extensively characterized the behavioral changes following concussion(s) at both acute and chronic time points (9). Using the same concussion paradigm in rats, Mountney et al observed that repeated concussions impaired motor function and produced gait abnormalities. It is possible that the early neuroinflammatory changes detected in the present study may have contributed to the development of behavioral dysfunction following repeated concussions in the PCI model.

Our study describes early changes in microglial phenotype and morphology following closed-head concussive injury in rats. Although pro-inflammatory M-1 phenotype dominates the early post-injury environment, a few M-2 markers were also elevated demonstrating that both types co-exist following injury. While a single concussion induced transient upregulation in inflammatory markers, multiple impacts exacerbated and sustained the response providing caution against sustaining multiple concussions while the brain is susceptible. Targeting microglial sub-types as opposed to reducing global microglial activation may provide a novel therapeutic approach for treating CHI.

## Disclosure

This material has been reviewed by the Walter Reed Army Institute of Research (WRAIR). There is no objection to its presentation and/or publication. The opinions or assertions contained herein are the private views of the author, and are not to be construed as official, or as reflecting true views of the Department of the Army, Department of Defense, the Uniformed Services University of the Health Sciences or any other agency of the U.S. Government.

## Author contributions

SM and BW designed the study. SM, BW, SU, WY, and LL performed data collection and data analysis. SM wrote the article. BW, SU, LL, JG, and DS contributed to data interpretation and critical evaluation of the manuscript.

### Conflict of interest statement

The authors declare that the research was conducted in the absence of any commercial or financial relationships that could be construed as a potential conflict of interest. The reviewer YZ declared a shared affiliation, though no other collaboration, with one of the authors LL to the handling Editor.
