# Supplementary material for: Early Microglial Activation Following Closed-Head Concussive Injury Is Dominated by Pro-Inflammatory M-1 Type
Source: Front Neurol. 2018 Nov 15;9:964. doi: 10.3389/fneur.2018.00964 (PMC6249371; doi:10.3389/fneur.2018.00964)
Supplement: Supplementary file 1 [file Table_1.docx]

| **Target Gene** | **TaqMan Assay ID** |
| --- | --- |
| Ccl2 | Rn00580555_m1 |
| Ccl3 | Rn01464736_g1 |
| IL-1b | Rn00580432_m1 |
| RT1-HA | Rn01768370_g1 |
| Arg1 | Rn00691090_m1 |
| CD36 | Rn00580728_m1 |
| Ccl22 | Rn01536591_m1 |
| Lif | Rn00573491_g1 |
| Iba-1 | Rn00574125_g1 |
| Sdha | Rn00590475_m1 |

**Supplementary Table-1:** Gene name and TaqMan IDs used for individual sample RT-PCR.
